# Circulation of HIV-1 CRF02_AG among MSM Population in Central Italy: A Molecular Epidemiology-Based Study

**DOI:** 10.1155/2013/810617

**Published:** 2013-11-28

**Authors:** Massimo Giuliani, Maria M. Santoro, Alessandra Lo Presti, Eleonora Cella, Paola Scognamiglio, Alessia Lai, Alessandra Latini, Lavinia Fabeni, Caterina Gori, Carmela Pinnetti, Enrico Girardi, Carlo F. Perno, Gianguglielmo Zehender, Massimo Ciccozzi

**Affiliations:** ^1^Department of Infectious Parasitic and Immunomediated Diseases, Istituto Superiore di Sanità, Rome, Italy; ^2^HIV/AIDS Unit, San Gallicano Dermatological Institute (IRCCS), Rome, Italy; ^3^University of Rome Tor Vergata, Rome, Italy; ^4^National Institute for Infectious Diseases L. Spallanzani, Rome, Italy; ^5^Department of Clinical Sciences, L. Sacco, University of Milan, Italy

## Abstract

*Introduction*. The evolutionary and demographic history of the circular recombinant form CRF02_AG in a selected retrospective group of HIV-1 infected men who have sex with men (MSM) resident in Central Italy was investigated. *Methods*. A total of 55 HIV-1 subtype CRF02_AG *pol* sequences were analyzed using Bayesian methods and a relaxed molecular clock to reconstruct their dated phylogeny and estimate population dynamics. *Results*. Dated phylogeny indicated that the HIV-1 CRF02_AG strains currently circulating in Central Italy originated in the early 90's. Bayesian phylogenetic analysis revealed the existence of a main HIV-1 CRF02_AG clade, introduced in the area of Rome before 2000 and subsequently differentiated in two different subclades with a different date of introduction (2000 versus 2005). All the sequences within clusters were interspersed, indicating that the MSM analyzed form a close and restricted network where the individuals, also moving within different clinical centers, attend the same places to meet and exchange sex. *Conclusions*. It was suggested that the HIV-1 CRF02_AG epidemic entered central Italy in the early 1990s, with a similar trend observed in western Europe.

## 1. Introduction

In the past decade, there has been an increase in the circulation of non-B strains and circulating recombinant forms (CRFs) of the human immunodeficiency virus type 1 (HIV-1) in previously subtype B homogeneous area such as Europe and North America [1–8]. In Europe, data from extensive multinational database showed that non-B strains and CRFs accounted for 15.0% to 30.0% of HIV-1 total infections in the different countries, with an increase in proportion from 2000 to 2007 [9–11].

Amongst the HIV-1 non-B types, the CRF02_AG is one of the most prevalent recombinant forms in the world, responsible for at least 8% of total infections [9]. Recently, its prevalence was increased in west and west-central Africa, where this viral variant is native and predominantly transmitted within heterosexual population, also in male at-risk populations, such as continental men who have sex with men (MSM) [10]. In western and central Europe, a similar trend was also observed, with an increased proportion of CRF02_AG-infected patients from 5% in 2000–2003 to 8% in 2004–2007 [9]. In Europe, this CRF represents the first HIV-1 non-B subtype among newly diagnosed individuals in many countries, such as Spain, Germany, Belgium, and The Netherlands [11].

In Italy, the proportion of HIV-1 non-B-infected patients has been progressively increased from 0.3% in 1993 to 20.0% in recent years [6, 12] and it exceeds 63.0% among foreigners living in this country [13–15]. Similarly in Lazio region a progressive increasing of the proportion of HIV-1 non-B-infected (from 6.1% in 2004 to 21.3% in 2009) has been observed among native Italian patients [16]. The high HIV-1 non-B subtypes prevalence in Italy can be associated to the increase of immigrants from Africa, Eastern Europe, and South America, accounted in the last decade [11, 16]. Among the HIV-1 non-B subtypes circulating in Italy, CRF02_AG is the second *per* proportion, after F and before C strains [6, 17–23].

To explore the increasing prevalence of the HIV-1 CRF02_AG and, in general, as those of other non-B subtypes, is important for epidemiological purposes but can also to be relevant in clinical setting. Some biological differences seem to exist between subtypes and this fact could have several implications in terms of development of drugs resistance, response to antiretroviral therapy, disease progression, and transmission rate.

Therefore, understanding of the reasons for the emergence and spread of non-B subtypes in certain core groups, such as MSM, is crucial for epidemiological analyses aimed to define the pathway of diffusion of HIV-1 nonnative strains in Europe over time and across the different sexual-risk populations.

In this regard, European MSM seem to have a high risk of infection with non-B subtypes because they are more likely than heterosexual individuals to have sex abroad, thus in countries with high proportions of non-B subtypes and with nonnational sexual partners [24, 25].

To improve knowledge about the spread of infection with the CRF02_AG, the evolutionary rates, and dates of origin of the epidemic were estimated and were identified and were characterized as a number of epidemiological clusters from a dataset of *pol* gene sequences isolated from MSM living in central Italy.

## 2. Materials and Methods

### 2.1. Study Population

Men who have sex with men (MSM) with confirmed HIV-1-infection by CRF02_AG were considered as eligible for the study. All the participants were identified from a large cohort of MSM newly diagnosed with HIV-1 infection from a surveillance network of clinical sites in the Lazio Region, from 2000 to 2012. Using a regional centralized archive, a set of *pol* sequences belonging to all participants was also selected. Thus, the study population represents all HIV-1 cases infected with a CRF02_AG subtype reported in the Lazio Region after the 2000 and for whom a *pol* sequence was available.

The dataset consisted of HIV-1 CRF02_AG *pol* sequences (1,191 nucleotides long). All the isolates were collected between 2000 and 2012. Demographic, behavioral, and clinical data of the selected patients were extracted from individual medical records and organized anonymously in a common electronic archive. All data was imputed, managed, and stored according to the standards and recommendations of the ethics committee of participant institutes.

### 2.2. HIV-1 *Pol* Sequencing

HIV genotype analysis was performed on plasma samples by means of a commercially available kit (ViroSeq HIV-1 genotyping system; Abbott Laboratories, Abbott Park, IL). Briefly, RNA was extracted, retrotranscribed by murine leukemia virus reverse transcriptase (RT), and amplified with Amplitaq-Gold polymerase enzyme by using 2 different sequence-specific primers for 40 cycles. *Pol*-amplified products (containing the entire protease and the first 335 amino acids of the RT open reading frame) were full length sequenced in sense and antisense orientations by an automated sequencer (ABI 3100) by using 7 different overlapping sequence-specific primers [26].

### 2.3. Statistical Analysis

To test the differences between Italian and nonnational individuals infected with HIV-1 CRF02-AG, the Mann-Whitney test for the continuous variables and Fisher's exact test for the categorical variables were used. *P*  values <0.05 were considered statistically significant. The calculations of all statistical tests were performed by using SPSS statistics package version 19.

### 2.4. Likelihood Mapping

The phylogenetic signal of our dataset was investigated by means of the likelihood mapping analysis of 10,000 random quartets by using TreePuzzle program as already described [27]. In this analysis, groups of four randomly chosen sequences (quartets) were evaluated using Maximum Likelihood (ML). For each quartet, the three possible unrooted trees were reconstructed under the selected substitution model. The posterior probabilities of each tree were then plotted on a triangular surface, so that fully resolved trees fall into the corners and the unresolved quartets in the centre of the triangle (indicating a star-like signal). When using this strategy, if more than 30% of the dots fall into the centre of the triangle, the data are considered unreliable for the purposes of phylogenetic inference.

### 2.5. Phylogenetic Analysis, Time-Scaled Phylogeny and Demographic History

All HIV *pol* sequences were aligned using Clustal X and manual editing was performed with Bioedit as already described [28], removing gaps and cutting to identical sequence lengths. ModelTest version. 3.7 was used to select the simplest evolutionary model that adequately fitted the sequence data [28].

Subtype was determined uploading sequences individually into the REGA HIV-1 automated Subtyping Tool version 2.0 (http://www.bioafrica.net/rega-genotype/html/subtypinghiv.html) and confirmed by in-house phylogenetic analysis. Maximum likelihood (ML) phylogenies were estimated with the best fitting nucleotide substitution model (GTR + I + G) (tested with ModelTest); calculations were performed with Phyml version 3.0. Statistical support for specific clades and clusters was assessed by bootstrap analysis considering bootstrap values >70% (data not shown).

The dated tree, evolutionary rates, and population growth were coestimated by using a Bayesian Markov Chain Monte Carlo approach (MCMC; Beast v. 1.7.4) implementing a general-time-reversible + invariant + gamma model. Different parametric demographic models (a constant population size, exponential and logistic growth) and a nonparametric Bayesian skyline plot (BSP) were compared under strict and relaxed clock conditions, and the best models were selected by means of a Bayes factor (BF, using marginal likelihoods) implemented in Beast as already described [28]. In accordance with Kass and Raftery [29], the strength of the evidence against H0 was evaluated as follows: 2lnBF <2; no evidence; 2–6: weak evidence: 6–10, strong evidence: >10, very strong evidence. A negative value indicates evidence in favor of H0. Only values of >6 were considered significant.

Chains were conducted for 30 × 10^6^ generations and sampled every 3,000 steps. Convergence of the MCMC was assessed on the basis of the effective sampling size (ESS) for each parameter. The effective sample size (ESS) of a parameter sampled from an MCMC (such as BEAST) is the number of effectively independent draws from the posterior distribution that the Markov chain is equivalent to. Only ESS values of >250 were considered robust and were accepted. Uncertainty in the estimates was indicated by 95% highest posterior density (95% HPD) intervals. The trees were summarized in a target tree by the Tree Annotator program included in the Beast package by choosing the tree with the maximum product of posterior probabilities (maximum clade credibility) after a 10% burn-in. The time of the most recent common ancestor (TMRCA) estimates was expressed as mean and 95% highest posterior density (HPD) years before the most recent sampling dates, corresponding to 2012 in this study.

The demographic history was also analyzed on the HIV-1 CRF02_AG *pol* sequences by performing the Bayesian skyline plot. The software MEGA 5 [30] allowed to calculate the mean genetic distances within different cluster in both subclades A and B by using Tamura Nei method.

## 3. Results

### 3.1. Patients' Characteristics

During the study period, 55 MSM infected by HIV-1 CRF02_AG were identified and reported by the network of clinical centers in the Lazio region. In particular, almost all of the participants (91.0%) were diagnosed at the National Institute of Infectious Diseases Lazzaro Spallanzani (Spallanzani, *n* = 31) and at the San Gallicano Dermatological Institute (San Gallicano, *n* = 19) in Rome. The other five patients were diagnosed at the San Giovanni Addolorata Hospital in Rome (San Giovanni *n* = 3), at the University of Tor Vergata in Rome (Tor Vergata *n* = 1) and at the Municipal Hospital in Latina (Latina *n* = 1). Forty-three patients (78.2%) were Italians. The median age was 33 years (IQR: 29–38).  Table 1 shows the selected demographic and clinical characteristics of selected patients. The analysis was performed on the overall population and by stratifying the patients by nationality. Sequences were collected in a median period of 2009 (IQR = 2007–2011). At the time of genotyping, the almost all of the patients (53/55, 96.4%) were drug-naïve. Median HIVRNA evaluation and CD4+ cell count were 4.8 (IQR = .3–5.4) log_10_ copies/mL and 445 × 10^6^ cells/l (IQR = 347–584), respectively. No significant differences were found between the Italians and nonnationals with respect to all the variables analyzed. The 12 nonnational individuals were originally from Brazil (*n* = 3), Cuba (*n* = 2), France (*n* = 1), Albania (*n* = 1), Latvia (*n* = 1), Spain (*n* = 1), Iceland (*n* = 1), Ecuador (*n* = 1), and Polynesia (*n* = 1).

### 3.2. Likelihood Mapping

The phylogenetic noise of our data set was investigated by means of likelihood mapping. The percentage of dots falling in the central area of the triangles was 6.3%, the likelihood map also showed the presence of 93.7% resolved quartets showing that the dataset alignment contained sufficient phylogenetic information (Figure  S1 in Supplementary Materials available on online at http://dx.doi.org/10.1155/2013/810617).

### 3.3. Phylogenetic Analysis, Time-Scaled Phylogeny Reconstruction and Demographic History

Phylogenetic analysis, conducted by the REGA HIV-1 automated subtyping tool confirmed that all the 55 HIV-1 sequences from participants are classifiable as CRF02_AG (data not shown). BF analysis showed that the relaxed clock fitted the data significantly better than the strict clock (2lnBF between the strict and relaxed clock was >200 in favor of the second). Under the relaxed clock, the BF analysis showed that the BSP was better than the other models (2lnBF > 48).

The estimated mean value of the HIV-1 CRF02_AG *pol *gene evolutionary rate was 2.17 × 10^−3^ substitution/site/year (95% HPD: 1.45 × 10^−3^–2.88 × 10^−3^).

**Figure 1:**
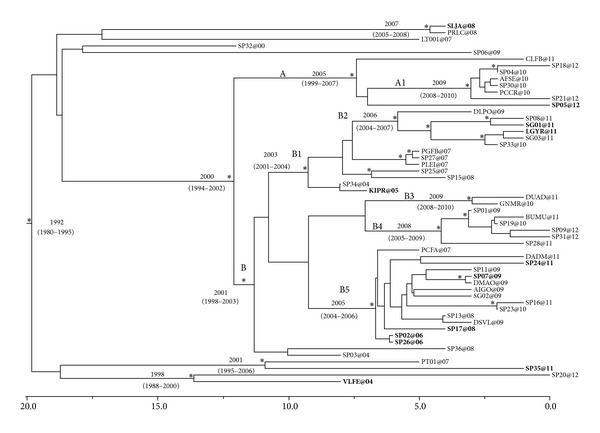
Bayesian time-scaled tree of the 55 HIV-1 CRF 02_AG *pol* sequences from MSM. The numbers at the root and at the internal nodes represent the estimated date of the origin and the uncertainty indicated by 95% highest posterior density (95% HPD) intervals. The asterisks (∗) along a branch represent significant statistical support for the clade subtending that branch (posterior probability >98%). Non-Italian sequences are in bold. The line at the bottom represents time (in years).

Figure 1 shows the Bayesian maximum clade credibility tree and the TMRCA estimates. The root of the tree had a TMRCA of 19.8 years corresponding to 1992 (95% HPD: 1980–1995). A main statistically supported clade, which dated back to 2000 (95% HPD: 1994–2002), was identified. Inside the main clade, a sub-clade A and a sub-clade B were evidenced, both statistically supported, and well defined for year of start of circulation. Specifically sub-clade A dated back to 2005 (95% HPD: 1999–2007). Inside sub-clade A, cluster A1 (which consisted of six of the eight isolates of sub-clade A) was statistically supported and dated back to 2009 (95% HPD: 2008–2010).

Sub-clade B had a TMRCA of 11 years corresponding to 2001 (95% HPD: 1998–2003). Inside sub-clade B, clusters B1, B2, B3, B4 and B5 were found. Cluster B1 included 13 sequences from patients who attended San Gallicano (*n* = 5), Spallanzani (*n* = 6) and San Giovanni Hospitals (*n* = 2), and dated back to 2003 (95% HPD: 2001–2004); within cluster B1, cluster B2 included six of the 13 sequences and dated back to the year 2006 (95% HPD: 2004–2007). Cluster B3 consisted of only two sequences and dated to 2009 (95% HPD: 2008–2010).

Cluster B4 was composed of six sequences (five of which were from patients who attended the Spallanzani Hospital) and had a TMRCA of four years corresponding to 2008 (95% HPD: 2005–2008).

Cluster B5 included 15 sequences (five from patients that were followed at San Gallicano Hospital, nine at Spallanzani Hospital and one at San Giovanni Hospital) and dated to the year 2005 (95% HPD: 2004–2006).

Outside the main clade, only three statistically supported groups were evidenced: the first one, which included the isolates labeled as SLJA@08 and PRLC@08 statistically supported, dated back to 2007 (95% HPD: 2005–2008); the second one included the isolates PT01@07 and SP35@11 and dated back to 2001 (95%HPD: 1995–2006); the third one included the isolates SP20@12 and VLFE@04 and dated back to 1998 (95%HPD: 1988–2000). Of note, sequences from nonnational and Italian MSMs did not form separate clusters but were rather interspersed within them.

**Figure 2:**
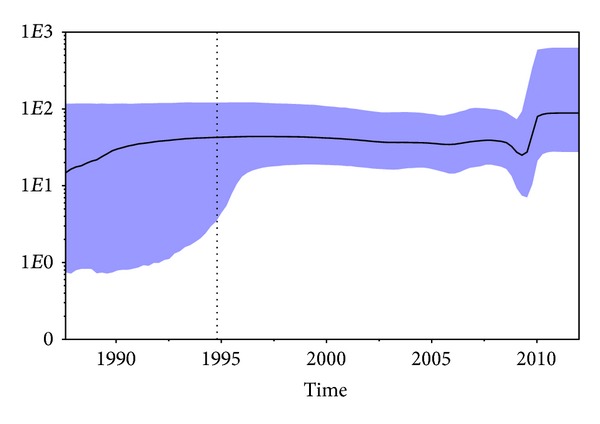
Bayesian skyline plot (BSP) of the 55 HIV-1 CRF 02_AG *pol* sequences from MSM. The effective number of infections is reported on the *Y*-axis. Time is reported in the *X*-axis. The colored area corresponds to the credibility interval based on 95% highest posterior density interval (HPD).

The demographic history of HIV-1 CRF02_AG subtype showed that the epidemic started in the end 80s and after a light grow until 1995 has remained more or less constant up to about 2007. After the 2007 the epidemic had a decrease, suffering of the bottleneck until 2010 when showed an increase over time (Figure 2).

The mean genetic distance was measured within clusters inside the subclades. The within groups mean genetic distance ranged from 1.6% for subclade B to 0.4% for subclade-A.

## 4. Discussion

MSM continue to be the population group at higher risk of acquiring HIV-1 infections in developed countries. In western Europe, the incidence of HIV-1 among MSM has increased over the last decade, probably due to an increase in unsafe risk sexual practices and the re-emergence of several sexually transmitted infections (STI) in the young, low educated and HIV unaware individuals [31–35]. The HIV epidemic in Italy started among intravenous drug users (i.e. heterosexual and MSM IDUs) who accounted for 57% of all the AIDS cases reported in the adult population between 1982 and 2007 and was mainly attributed to the HIV-1 B subtype [36]. In Europe, national surveillance data showed an increasing proportion of HIV-1 cases among MSM, ranging from 15% in 1996-1997 to 22% in 2006-2007 [31]. In Italy, HIV-1 CRF02_AG accounts for about 3% of all the infected and represents 80% or more of all the CRFs in the country [37].

The aim of this study was to investigate the evolutionary and demographic history of this CRF in a selected retrospective group of HIV-1 infected MSM resident in central Italy, by using a Bayesian coalescent-based framework. Dated phylogeny allowed to estimate a TMRCA in 1992 for the root of the tree, thus suggesting that the HIV-1 CRF02_AG strains currently circulating in Central Italy originated in the early 90's and that the epidemic was relatively close to the HIV-1 B subtype introduction in the 80s [38]. This is the first molecular epidemiology-based study in Italy which aimed to investigate the characteristics and timing of circulation of CRF02_AG in HIV-1 infected people, in particular in MSM.

Bayesian phylogenetic analysis on the 55 *pol* sequences revealed the existence of a main clade statistically supported, introduced in Rome before 2000 and differentiated over time into two sub-clades (A and B) well defined for year of start of circulation (2000 and 2005). Inside the sub-clade A, we found only one cluster (A1) statistically supported, whereas inside the sub-clade B we identified five different statistically supported clusters (B1 to B5).

All sequences within identified cluster were interspersed, indicating that the subgroup of MSM analyzed in this study belongs to a close and restricted network of individuals, which despite attending different clinical centers attend the same place for engagement and sexual exchanges. This hypothesis is also in line with the fact that the only sequence found in the MSM living in Latina, Southern Lazio, did not cluster with any of the sequences retrieved among MSM living in Rome, probably because MSM in Latina hang around different meeting places than those living in Rome. Conversely, sequences were relatively dispersed to several clinical centers, presumably due to a variety of factors, which commonly influence clinical facilities from general population, such as recommendations from family, friends and neighbours, trust in a given physician, and closeness of specific clinical centers to own house. The presence of so many different clusters in the phylogeny also indicated that different viral introductions occurred at different times, as expected for this young epidemic. A number of authors have recently attempted the timescale reconstruction and characterization of epidemiological clusters among individuals infected with HIV-1 (particularly MSM) using molecular clock-based approaches [39, 40]. Hué et al. identified in United Kingdom (UK) different clusters of HIV-1 B subtypes concluding that different epidemics are involved the introduction of this subtype in MSM as we observed from our data. Although the UK cohort was bigger than our group of MSM, the peculiarity of our dataset is that it was obtained from a restricted and homogeneous group of MSM, mostly living in Rome and all attending clinical centers within the same metropolitan area. In the light of all these considerations, we can hypothesize that all individuals analyzed in the present study were infected by the same HIV-1 CRF02_AG. All the clusters within clades A and B, ranging from 2003 to 2009, had a low level of intraclade genetic heterogeneity, as shown by the different genetic distance, suggesting that these strains probably did not accumulate a large number of mutations over time. This hypothesis is supported by the positive correlation between the TMRCA estimates and the genetic diversity (ranging from 1.6 to 0.4%) observed over the time. Because Italians, and nonnational MSM did not form separate clusters but were instead rather interspersed within the same cluster, it was not possible to establish who infected whom. Probably some nonnational individuals arrived in Italy are already infected, while others did not.

We did not find any significantly supported clades before 2000, probably because of the high rate of mortality among HIV-infected subjects before the introduction of highly active antiretroviral therapy (HAART); moreover, we should remember that our samples were collected between 2000 and 2012 [38]. Usually, one possible bias in the identification of epidemiological clusters is represented by the effects of convergent evolution due to drug therapy-associated selection pressure. Our demographic analysis by logistic model showed that the epidemic grew in Italy, which is to a certain extent supported by other authors findings about subtype B epidemic spread in high-income countries [41]. Indeed, the BSP showed that the epidemic was characterized by a slight exponential growth from the first half of 1990s (corresponding to the root of the tree) until the early 1995s. During this period, the effective number of infections increased by the low rate of 0.5 cases *x* year [42]. After a constant grows the viral population experienced a bottle neck and a the number of cases decreased until 2010, probably because of the death of infected individuals in the community, but again increased after this date reaching a sort of plateau. This result is largely consistent with what happened among MSM over western Europe, where the HIV-1 upraise in the early 2000s was preceded by years of continuous but slow decline in the number of HIV-1 being diagnosed [33–35, 43]. Moreover, the recent replacement of the endemic subtype by new subtypes from other geographic areas [44, 45] may partly explain the decrease in the number of people infected with HIV-1 B subtype, but the epidemiological analysis did not show any trend in the recent clades which may support an increased proportion of cases in the non-Nationals. Therefore, we cannot suggest that a relation with a possible recent introduction of this recombinant form exists. However, the relatively high number of nonnationals (about 22%) analyzed in our group of MSM can ring alarm bell suggesting a significant spillover of CRF02_AG into the Italian population fostered by the steady increase of immigrants from different African countries during the past decade. Given the position of Italy in the Mediterranean Sea as a strategic travel route between Africa and western Europe, HIV-1 molecular epidemiology may change over time. Understanding the CRF02_AG epidemic from Africa to Italy may also play a fundamental role in assessing the potential spread of this viral strain within western Europe, given the vast exchange of persons and goods between southern and western Europe. Unfortunately, no consistent data from other similar national studies were available also to make useful comparisons with the same population of MSM or other ones.

Understanding HIV molecular epidemiology and potential future spread of different non-B subtypes has also clinical relevance. It is already known that differences among HIV-1 genetic forms may impact both the clinical management and surveillance of drug resistance, as a result of the effect of treatment on non-B HIV-1 strains [45, 46].

Moreover, HIV-1 subtypes must be considered in the vaccine development process [45, 47]. Although cross-clade immune reactivity has been detected among individuals and vaccine recipients, it is reasonable to expect that a vaccine with an antigenic composition including CRFs may produce a more effective response [45].

## Supplementary Material

Supplementary Figure: Likelihood mapping of 55 HIV-1 CRF 02)_AG *pol* sequences from MSM . The dots inside the triangles represent the posterior probabilities of the possible unrooted topologies for each quartet. Numbers indicate the percentage of dots in the centre of the triangle corresponding to phylogenetic noise (star-like trees).

## Figures and Tables

**Table 1:** Patients' characteristics at the moment of genotypic request.

Variables	Overall	Italians	Nonnationals	*P* value
Number of patients	55	43	12	
Age (years), median (IQR)	33 (29–38)	32 (29–38)	34 (30–38)	0.610
Viremia (log_10_⁡ copies/mL), median (IQR)	4.8 (4.3–5.4)	4.8 (4.3–5.3)	5.0 (4.3–5.6)	0.441
CD4+ (10^6^ cells/L), median (IQR)	445 (347–584)	444 (336–589)	445 (371–556)	0.761
Year of first seropositivity, median (IQR)	2009 (2007–2011)	2009 (2007–2011)	2009 (2006–2011)	0.813
Year of genotyping, median (IQR)	2009 (2007–2011)	2009 (2008–2011)	2009 (2006–2011)	0.557
Treatment status, *n* (%):				
Naïve	53 (96.4)	41 (95.3)	12 (100.0)	1
Treatment interruption	2 (3.6)	2 (4.7)	0 (0.0)	

IQR: interquartile range.

## References

[1] Barin F, Courouce A-M, Pillonel J, Buzelay L (1997). Increasing diversity of HIV-1(M) serotypes in French blood donors over a 10-year period (1985–1995). *AIDS*.

[2] Weidle PJ, Ganea CE, Irwin KL (2000). Presence of human immunodeficiency virus (HIV) type 1, group M, non-B subtypes, Bronx, New York: a sentinel site for monitoring HIV genetic diversity in the United States. *Journal of Infectious Diseases*.

[3] Yerly S, Vora S, Rizzardi P (2001). Acute HIV infection: impact on the spread of HIV and transmission of drug resistance. *AIDS*.

[4] Frange P, Galimand J, Vidal N (2008). New and old complex recombinant HIV-1 strains among patients with primary infection in 1996–2006 in France: the French ANRS CO06 Primo Cohort Study. *Retrovirology*.

[5] Holguín A, de Mulder M, Yebra G, López M, Soriano V (2008). Increase of non-B subtypes and recombinants among newly diagnosed HIV-1 native spaniards and immigrants in Spain. *Current HIV Research*.

[6] Lai A, Riva C, Marconi A (2010). Changing patterns in HIV-1 non-B clade prevalence and diversity in Italy over three decades. *HIV Medicine*.

[7] Brennan CA, Yamaguchi J, Devare SG, Foster GA, Stramer SL (2010). Expanded evaluation of blood donors in the United States for human immunodeficiency virus type 1 non-B subtypes and antiretroviral drug-resistant strains: 2005 through 2007. *Transfusion*.

[8] Carr JK, Osinusi A, Flynn CP, Gilliam BL, Maheshwari V, Zhao RY (2010). Two independent epidemics of HIV in Maryland. *Journal of Acquired Immune Deficiency Syndromes*.

[9] Hemelaar J, Gouws E, Ghys PD, Osmanov S (2011). Global trends in molecular epidemiology of HIV-1 during 2000–2007. *AIDS*.

[10] Ndiaye HD, Tchiakpe E, Vidal N (2013). HIV type 1 subtype C remains the predominant subtype in men having sex with men in senegal. *AIDS Research and Human Retroviruses*.

[11] Abecasis B, Wensing AMJ, Paraskevis D (2013). HIV-1 subtype distribution and its demographic determinants in newly diagnosed patients in Europe suggest highly compartmentalized epidemics. *Retrovirology*.

[12] Ciccozzi M, Santoro MM, Giovanetti M, Andrissi L, Bertoli A, Ciotti M (2012). HIV-1 non-B subtypes in Italy: a growing trend. *The New Microbiologica*.

[13] Balotta C, Bagnarelli P, Riva C (1997). Comparable biological and molecular determinants in HIV type 1-infected long-term nonprogressors and recently infected individuals. *AIDS Research and Human Retroviruses*.

[14] Ciccozzi M, Montieri S, Salemi M (2007). An outbreak of HIV-1 subtype G among Italian injecting drug users. *AIDS*.

[15] Buonaguro L, Tagliamonte M, Tornesello ML (2004). Screening of HIV-1 isolates by reverse heteroduplex mobility assay and identification of non-B subtypes in Italy. *Journal of Acquired Immune Deficiency Syndromes*.

[16] Navarra A, Orchi N, Palummieri A Increasing trend in non-B subtypes among Italians newly diagnosed with HIV in Rome, 2004–2009.

[17] Santoro MM, Alteri C, Ronga L (2012). Comparative analysis of drug resistance among B and the most prevalent non-B HIV type 1 subtypes (C, F, and CRF02_AG) in Italy. *AIDS Research and Human Retroviruses*.

[18] Monno L, Brindicci G, Lo Caputo S (2005). HIV-1 subtypes and Circulating Recombinant Forms (CRFs) from HIV-infected patients residing in two regions of central and southern Italy. *Journal of Medical Virology*.

[19] Tramuto F, Bonura F, Perna AM (2007). Genetic diversity of HIV-1 non-B strains in Sicily: evidence of intersubtype recombinants by sequence analysis of gag, *pol*, and env genes. *AIDS Research and Human Retroviruses*.

[20] Bracciale L, Colafigli M, Zazzi M (2009). Prevalence of transmitted HIV-1 drug resistance in HIV-1-infected patients in Italy: evolution over 12 years and predictors. *Journal of Antimicrobial Chemotherapy*.

[21] Giuliani M, Montieri S, Palamara G (2009). Non-B HIV type 1 subtypes among men who have sex with men in Rome, Italy. *AIDS Research and Human Retroviruses*.

[22] Riva C, Lai A, Caramma I (2010). Transmitted HIV type 1 drug resistance and non-B subtypes prevalence among seroconverters and newly diagnosed patients from 1992 to 2005 in Italy. *AIDS Research and Human Retroviruses*.

[23] Torti C, Lapadula G, Izzo I (2010). Heterogeneity and penetration of HIV-1 non-subtype B viruses in an Italian province: public health implications. *Epidemiology and Infection*.

[24] Elford J, Bolding G, Davis M, Sherr L, Hart G (2004). Web-based behavioral surveillance among men who have sex with men: a comparison of online and offline samples in London, UK. *Journal of Acquired Immune Deficiency Syndromes*.

[25] Mercer CH, Fenton KA, Wellings K, Copas AJ, Erens B, Johnson AM (2007). Sex partner acquisition while overseas: results from a British national probability survey. *Sexually Transmitted Infections*.

[26] Ceccherini-Silberstein F, Erba F, Gago F (2004). Identification of the minimal conserved structure of HIV-1 protease in the presence and absence of drug pressure. *AIDS*.

[27] Zehender G, Ebranati E, Bernini F (2011). Phylogeography and epidemiological history of West Nile virus genotype 1a in Europe and the Mediterranean basin. *Infection, Genetics and Evolution*.

[28] Ciccozzi M, Madeddu G, Lo Presti A (2013). HIV type 1 origin and transmission dynamics among different risk groups in sardinia: molecular epidemiology within the close boundaries of an Italian island. *AIDS Research and Human Retroviruses*.

[29] Kass RE, Raftery AE (1995). Bayes factors. *Journal of the American Statistical Association*.

[30] Tamura K, Peterson D, Peterson N, Stecher G, Nei M, Kumar S (2011). MEGA5: molecular evolutionary genetics analysis using maximum likelihood, evolutionary distance, and maximum parsimony methods. *Molecular Biology and Evolution*.

[31] UNAIDS/WHO Report on the Global HIV/AIDS Epidemic 2008: Executive Summary. http://data.unaids.org/pub/GlobalReport/2008/JC1511_GR08_ExecutiveSummary_en.pdf.

[32] Hamers FF, Downs AM (2004). The changing face of the HIV epidemic in western Europe: what are the implications for public health policies?. *The Lancet*.

[33] Jansen IAV, Geskus RB, Davidovich U (2011). Ongoing HIV-1 transmission among men who have sex with men in Amsterdam: a 25-year prospective cohort study. *AIDS*.

[34] Semaille C, Cazein F, Lot F (2009). Recently acquired HIV Infection in men who have sex with men (MSM) in France, 2003–2008. *Euro Surveillance*.

[35] Giuliani M, Di Carlo A, Palamara G (2005). Increased HIV incidence among men who have sex with men in Rome. *AIDS*.

[36] Suligoi B, Camoni L, Boros S (2012). Aggiornamento delle nuove diagnosi da HIV e dei casi di AIDS in Italia al 31 dicembre 2011. *Notiziario Dell'Istituto Superiore di Sanità*.

[37] HIV DATABASE http://www.hiv.lanl.gov/content/index.

[38] Walker PR, Pybus OG, Rambaut A (2005). Comparative population dynamics of HIV-1 subtypes B and C: subtype-specific differences in patterns of epidemic growth. *Infection, Genetics and Evolution*.

[39] Hué S, Pillay D, Clewley JP, Pybus OG (2005). Genetic analysis reveals the complex structure of HIV-1 transmission within defined risk groups. *Proceedings of the National Academy of Sciences of the United States of America*.

[40] Lewis F, Hughes GJ, Rambaut A, Pozniak A, Leigh Brown AJ (2008). Episodic sexual transmission of HIV revealed by molecular phylodynamics. *PLoS Medicine*.

[41] Bello G, Eyer-Silva WA, Couto-Fernandez JC (2007). Demographic history of HIV-1 subtypes B and F in Brazil. *Infection, Genetics and Evolution*.

[42] de Oliveira T, Deforche K, Cassol S (2005). An automated genotyping system for analysis of HIV-1 and other microbial sequences. *Bioinformatics*.

[43] Phillips AN, Cambiano V, Nakagawa F (2013). Increased HIV incidence in men who have sex with men despite high levels of ART-induced viral suppression: analysis of an extensively documented epidemic. *PLOS ONE*.

[44] Buonaguro L, Tagliamonte M, Tornesello ML (2007). Genetic and phylogenetic evolution of HIV-1 in a low subtype heterogeneity epidemic: the Italian example. *Retrovirology*.

[45] Thomson MM, Nájera R (2001). Travel and the introduction of human immunodeficiency virus type 1 non-B subtype genetic forms into Western countries. *Clinical Infectious Diseases*.

[46] Easterbrook PJ, Smith M, Mullen J (2010). Impact of HIV-1 viral subtype on disease progression and response to antiretroviral therapy. *Journal of the International AIDS Society*.

[47] Peeters M, Toure-Kane C, Nkengasong JN (2003). Genetic diversity of HIV in Africa: impact on diagnosis, treatment, vaccine development and trials. *AIDS*.

