# Supplementary material for: Circulation of HIV-1 CRF02_AG among MSM Population in Central Italy: A Molecular Epidemiology-Based Study
Source: Biomed Res Int. 2013 Nov 28;2013:810617. doi: 10.1155/2013/810617 (PMC3863479; doi:10.1155/2013/810617)
Supplement: Supplementary file 1 — Supplementary Figure: Likelihood mapping of 55 HIV-1 CRF 02)_AG pol sequences from MSM . The dots inside the triangles represent the posterior probabilities of the possible unrooted topologies for each quartet. Numbers indicate the percentage of dots in the centre of the triangle corresponding to phylogenetic noise (star-like trees). [file 810617.f1.pdf]

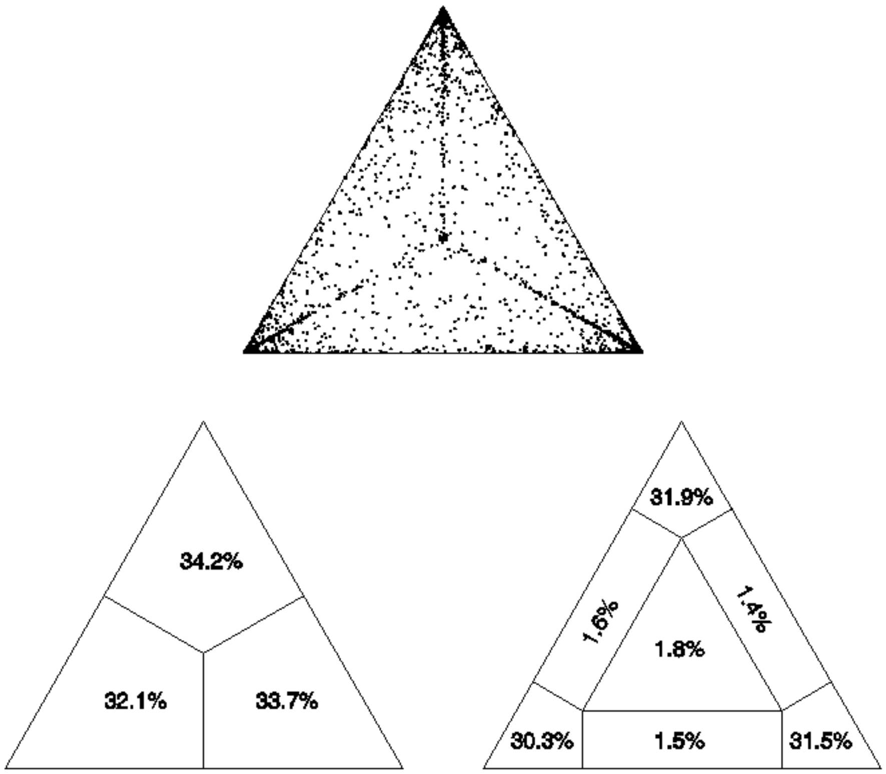

Supplementary Figure 1. Likelihood mapping of 55 HIV-1 CRF 02_ AG *pol* sequences from MSM . The dots inside the triangles represent the posterior probabilities of the possible unrooted topologies for each quartet. Numbers indicate the percentage of dots in the centre of the triangle corresponding to phylogenetic noise (star-like trees).
